# The accumulation mechanism of the hypoxia imaging probe “FMISO” by imaging mass spectrometry: possible involvement of low-molecular metabolites

**DOI:** 10.1038/srep16802

**Published:** 2015-11-19

**Authors:** Yukiko Masaki, Yoichi Shimizu, Takeshi Yoshioka, Yukari Tanaka, Ken-ichi Nishijima, Songji Zhao, Kenichi Higashino, Shingo Sakamoto, Yoshito Numata, Yoshitaka Yamaguchi, Nagara Tamaki, Yuji Kuge

**Affiliations:** 1 Shionogi Innovation Center for Drug Discovery, Discovery Research Laboratory for Innovative Frontier Medicines, Shionogi & Co., Ltd., Sapporo 001-0021, Japan; 2 Central Institute of Isotope Science, Hokkaido University, Sapporo 060-0815, Japan; 3 Graduate School of Medicine, Hokkaido University, Sapporo 060-8638, Japan; 4 Faculty of Pharmaceutical Sciences, Hokkaido University, Sapporo 060-0812, Japan; 5 Shionogi Pharmaceutical Research Center, Research Laboratory for Development, Shionogi & Co., Ltd., Osaka 561-0825, Japan

## Abstract

^18^F-fluoromisonidazole (FMISO) has been widely used as a hypoxia imaging probe for diagnostic positron emission tomography (PET). FMISO is believed to accumulate in hypoxic cells via covalent binding with macromolecules after reduction of its nitro group. However, its detailed accumulation mechanism remains unknown. Therefore, we investigated the chemical forms of FMISO and their distributions in tumours using imaging mass spectrometry (IMS), which visualises spatial distribution of chemical compositions based on molecular masses in tissue sections. Our radiochemical analysis revealed that most of the radioactivity in tumours existed as low-molecular-weight compounds with unknown chemical formulas, unlike observations made with conventional views, suggesting that the radioactivity distribution primarily reflected that of these unknown substances. The IMS analysis indicated that FMISO and its reductive metabolites were nonspecifically distributed in the tumour in patterns not corresponding to the radioactivity distribution. Our IMS search found an unknown low-molecular-weight metabolite whose distribution pattern corresponded to that of both the radioactivity and the hypoxia marker pimonidazole. This metabolite was identified as the glutathione conjugate of amino-FMISO. We showed that the glutathione conjugate of amino-FMISO is involved in FMISO accumulation in hypoxic tumour tissues, in addition to the conventional mechanism of FMISO covalent binding to macromolecules.

Hypoxia, or low oxygen concentration, in tumours has emerged as an important factor promoting tumour progression, angiogenesis and resistance to radiotherapy and chemotherapy^1^,^2^. Therefore, early identification of the location and extent of hypoxia is essential to the clinical management of cancer. To achieve this, non-invasive detection of hypoxic areas within tumours has been attempted with several molecular imaging technologies^3^. Among these modalities, positron emission tomography (PET) is a non-invasive diagnostic imaging technique for measuring biological activity with great sensitivity and quantitative accuracy^4^.

For hypoxia imaging with PET, various agents have been developed. Most of these compounds contain a 2-nitroimidazole structure, because it is well known that 2-nitroimidazole derivatives are reduced and specifically accumulate in hypoxic areas^5^. ^18^F-fluoromisonidazole (FMISO), an ^18^F-labelled 2-nitroimidazole derivative, is the most widely used hypoxia-imaging probe for PET diagnosis^6^. FMISO is believed to bind covalently to macromolecules in hypoxic cells after reduction of its nitro group (Fig. 1)^3^. However, the detailed mechanism of its accumulation remains unknown. This is mainly because conventional radiological imaging techniques including autoradiography (ARG) and PET show only the distribution of radioactivity without providing structural information of the labelled agent. Accordingly, using these methods, it is impossible to differentially image the distributions of the radiolabelled agent and its metabolites in tissues.

Imaging mass spectrometry (IMS) was developed to directly visualise distribution of molecules on tissue sections^7^. Over the past few years, this technique has been widely used to investigate distribution of molecules such as peptides, lipids, drugs and endogenous metabolites^8^,^9^,^10^. Because it uses MS-based detection, IMS can evaluate numerous molecules in a single measurement without a specialised probe. This property enables it to distinguish among distributions of a drug and its metabolites on tissue sections^11^. Therefore, IMS has the potential to be an effective imaging technique for drug distribution measurements.

In this study, we employed a combination of radioisotope analysis and IMS to elucidate the mechanism of FMISO accumulation in hypoxic tumour tissues.

## Results

### Biodistribution study

The biodistribution of ^18^F-FMISO in tumour-bearing mice is shown in Fig. S1. Higher radioactivity accumulation was observed in tumours as compared with blood and muscle. The ratio of radioactivity levels in tumour to those in blood was 1.43 ± 0.50 and 1.32 ± 0.12 at 2 and 4 h, respectively. The equivalent ratio in tumour to muscle was 1.31 ± 0.52 and 1.12 ± 0.30 at 2 and 4 h, respectively.

### Metabolite analysis of radiolabelled FMISO in tumour tissues

The distribution of radioactivity covalently bound to macromolecules versus unbound was determined by methanol extraction (Fig. 2A). Extracted and unextracted fractions of total radioactivity were interpreted as being low molecular weight and covalently bound to macromolecules, respectively. Using this assessment, the percent radioactivity covalently bound to macromolecules was 32.2 ± 4.0% (n = 4) in tumour homogenates.

To characterise the low-molecular-weight fraction, radio-high-performance liquid chromatography (HPLC) analysis was performed (Fig. 2B), detecting unmodified FMISO as well as amino-FMISO, in which the nitro group in the imidazole ring of FMISO had been reduced to an amino group. The percentages of these two species in the low-molecular-weight fraction were about 20% and 5%, respectively. This result unexpectedly suggested that those species, especially amino-FMISO, do not contribute substantially to the ARG images of the tumour derived from the FMISO-injected mice. In addition to these molecules, fractions whose chemical structures could not be identified constituted the largest portion of the visible radioactivity (45%).

### Distribution of radioactivity in tumour sections from ^18^F-FMISO-injected mice

To evaluate the distributions of FMISO covalently bound to macromolecules, ARG was performed, with and without washing, on tumour sections from ^18^F-FMISO-injected mice (Fig. 2C,D). The distribution pattern acquired from the washed sections corresponded to that from the unwashed sections. However, washing substantially decreased the total radioactivity.

### Distribution of FMISO and its derivatives in tumours

To evaluate distributions of FMISO and amino-FMISO, IMS analysis was performed using ultrahigh resolution Fourier transform-ion cyclotron resonance (FTICR)-MS.

This analysis revealed that both unmodified FMISO (Fig. 3A) and amino-FMISO (Fig. 3B) were nonspecifically distributed in tumours, with distribution patterns that did not correspond to those observed by ARG (Fig. 3C) or pimonidazole immunohistochemistry measurements (Fig. 3D) on serial sections.

In addition, peaks with the same m/z as reductive intermediates of FMISO were detected. These m/z values were 174.067 for nitroso-FMISO (Fig. 4A) and 176.083 for hydroxylamino-FMISO (Fig. 4B). These are expected intermediates in the reduction of FMISO to amino-FMISO. Similar to the observations with FMISO (Fig. 3A) and amino-FMISO (Fig. 3B), the distributions of these intermediates (Fig. 4A,B) were nonspecific in the tumour and did not correspond to distributions of radioactivity observed with ARG (Fig. 4D) and positive pimonidazole immunohistochemical staining (Fig. 4E).

Exploring molecules contained specifically in the FMISO-dosed tumours with an MS-based search, we found a molecule with m/z 465.157 whose distribution pattern corresponded to that of the radioactivity in the ARG images (Fig. 4C). Its distribution was also correlated with positive pimonidazole immunohistochemical staining.

To validate the identity of this newly discovered molecule, isotope pattern analysis was applied to the detected peak in tumour sections (Fig. 5B). Both the accurate mass and isotope pattern detected by FTICR-MS with ultrahigh resolution agreed with theoretical values. This supported the identity of this molecule as the hypothesised glutathione conjugate of amino-FMISO.

The presence of a glutathione conjugate of amino-FMISO in the tumours was confirmed by liquid chromatography-tandem mass spectrometry (LC-MS/MS) (Fig. 5C). A chemical structure and potential fragmentation pattern of the glutathione conjugate of amino-FMISO are shown in Fig. 5A. Multiple fragment peaks detected by LC-MS/MS analysis agreed with predictions^12^.

In the low-molecular-weight fraction, the amount of FMISO and amino-FMISO and semi-quantitative values of FMISO-derived material that was glutathione-conjugated were evaluated by LC-MS/MS (Fig. S2). Similar to observations of radioactivity in the low-molecular-weight fraction, the amounts of FMISO and amino-FMISO at 4 h after dosing were lower than at 2 h. However, during the same time period the ion intensity of glutathione conjugated amino-FMISO was increased.

## Discussion

In our study, we found that most of the radioactivity derived from ^18^F-FMISO existed as low-molecular-weight substances in the tumours and these were strongly reflected in the ARG images. Our IMS study revealed that a low-molecular-weight FMISO metabolite, the glutathione conjugate of its reduced form, amino-FMISO, was a component responsible for FMISO accumulation in the hypoxic regions of tumours.

**Figure 1:**
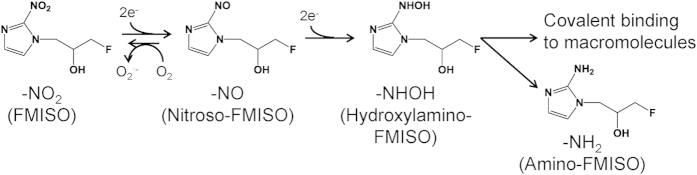
Proposed mechanism of reduction and accumulation of FMISO in hypoxic tissue regions.

**Figure 2:**
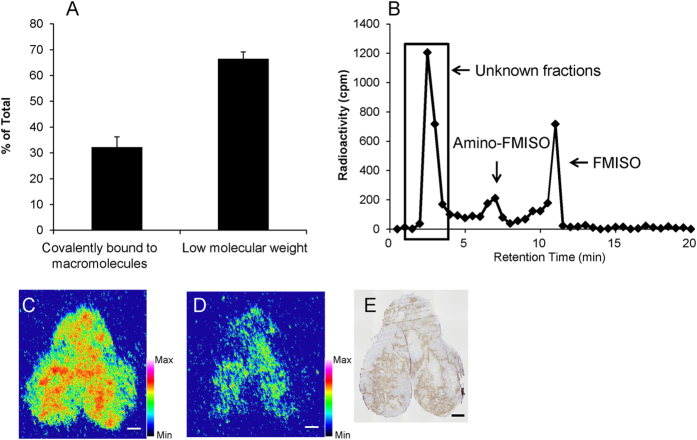
Distribution of radioactivity in tumours derived from ^18^F-FMISO injected mice. (**A**) Distribution of radioactivity from ^18^F-FMISO between a fraction covalently bound to macromolecules and a low-molecular-weight fraction. Data are means ± s.d. (n = 3). (**B**) Radio-HPLC chromatogram of the low-molecular-weight fraction of FMISO. (**C**,**D**) Autoradiograph (ARG) of tissue sections without (**C**) and with (**D**) washing. Scale bar represents 1 mm. (**E**) Immunohistochemical staining for pimonidazole. Scale bar represents 1 mm.

To estimate ^18^F-FMISO-derived compounds in the tumours, we performed solvent extraction and radio-HPLC analysis. In this manner, we found that most of the radioactivity derived from ^18^F-FMISO existed as low-molecular-weight species (Fig. 2A) and that the levels of unmodified FMISO and reduced FMISO (amino-FMISO, Fig. 1) were relatively low (Fig. 2B). These results indicated that most of the radioactivity corresponded to unknown compounds (Fig. 2B). Furthermore, we performed ARG studies on tumour sections from ^18^F-FMISO-treated mice, with or without washing of the sections in hydrophilic solvents (Fig. 2C,D). The ARG images of the washed sections are intended to show material covalently bound to macromolecules. This is because ^18^F-FMISO-derived low-molecular-weight compounds are relatively hydrophilic and should be easily removed by solvents such as tris-buffered saline and ethanol. The correspondence of the ARG image, with washing, to positive staining for pimonidazole (Fig. 2E) suggests that covalent binding to macromolecules contributes to the distribution of ^18^F-FMISO-derived radioactivity in tumour hypoxic regions. This is the mechanism that has been conventionally proposed^13^. However, most of the radioactivity observed without washing was removed by washing. This indicates that ^18^F-FMISO-derived low-molecular-weight compounds contribute substantially to the specific localisation of radioactivity within the tumour tissues. This is consistent with our finding that most of the ^18^F-FMISO-derived radioactivity in tumour tissues existed as low-molecular-weight substances. In addition, a considerable portion of radioactivity in these tumours was found to be unidentified compounds by radio-HPLC analysis. Therefore, it is suggested that the ARG images primarily reflected distribution of these unknown low-molecular-weight compounds.

**Figure 3:**
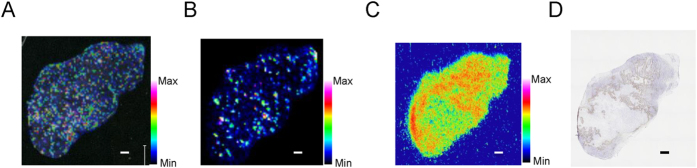
Representative mass spectrometric images of FMISO and amino-FMISO, ARG and pimonidazole staining in mouse tumour 4 h after administration of ^18^F-FMISO. Scale bar represents 1 mm. (**A**) Mass spectrometric images of m/z 190->m/z 174.0673 representing FMISO. (**B**) Mass spectrometric images of m/z 160.088 representing amino-FMISO. (**C**) ARG image showing total radioactivity. (**D**) Immunohistochemical staining for pimonidazole.

**Figure 4:**
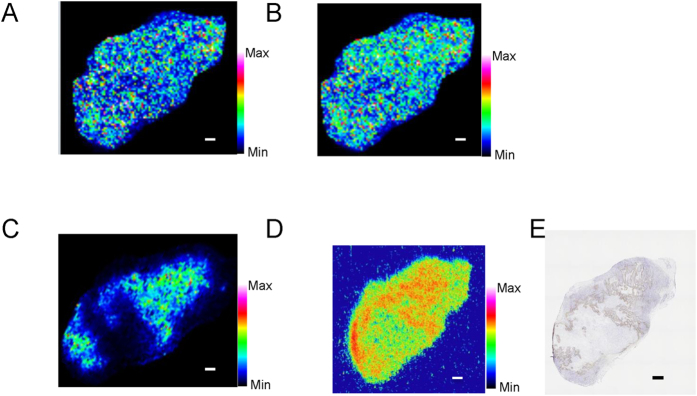
Representative mass spectrometric images of low-molecular-weight FMISO metabolites, ARG and pimonidazole staining in mouse tumour 4 h after administration of ^18^F-FMISO. Scale bar represents 1 mm. (**A**) Mass spectrometric images of m/z 174.067 representing nitroso-FMISO (a reductive intermediates of FMISO, see Fig. 1). (**B**) Mass spectrometric images of m/z 176.083 representing hydroxylamino-FMISO (a reductive intermediates of FMISO, see Fig. 1). (**C**) Mass spectrometric images of m/z 465.157 representing glutathione conjugate of amino-FMISO. (**D**) ARG showing total radioactivity. (**E**) Immunohistochemical staining for pimonidazole.

**Figure 5:**
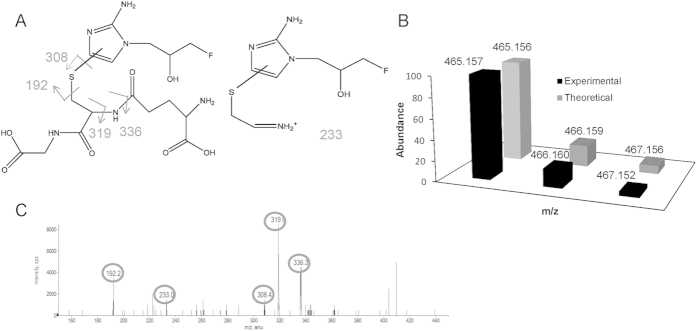
Validation of the glutathione conjugate of amino-FMISO in mouse tumour by isotope pattern and MS/MS analysis. (**A**) Structure and predicted MS/MS pattern of glutathione conjugate of amino-FMISO. (**B**) Isotope pattern of glutathione conjugate of amino-FMISO. (**C**) Fragmentation pattern from MS/MS analysis of m/z 465.157 in mouse tumour

**Figure 6:**
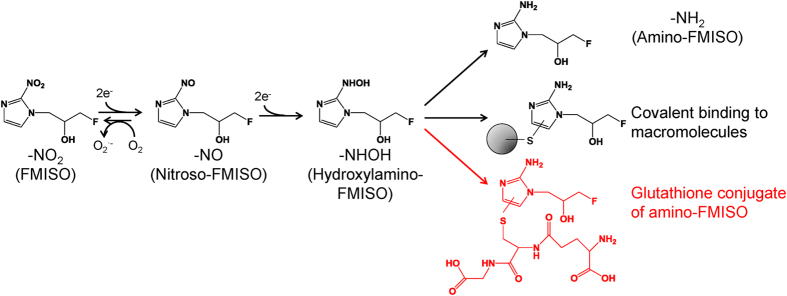
Proposed mechanism of metabolism and tissue accumulation of FMISO in tumour hypoxic regions revealed from this study.

Our IMS study showed that the expected FMISO metabolites, namely amino-FMISO and reductive intermediates of FMISO (Fig. 1), in addition to unmodified FMISO, were diffusely distributed in the tumours. These distribution patterns did not correspond to those observed in ARG images or by immunohistochemical staining for pimonidazole (Figs 3 and 4). This suggests that unmodified FMISO and the predicted FMISO metabolites were not localised in the hypoxic areas of the tumours and did not contribute to the specific distribution of ^18^F-FMISO-derived radioactivity observed by ARG. Therefore, we postulated that there were other molecules primarily responsible for the distribution pattern detected in ARG images. To elucidate the nature of these compounds, we performed an IMS search enabling simultaneous detection of potential metabolites by broadening the scope of the IMS measurements, comparing mass spectra of tumour sections from FMISO-treated and untreated mice. We found that the distribution pattern of the glutathione conjugate of amino-FMISO corresponded well to that of radioactivity in ARG images (Fig. 4). In addition, the amount of glutathione conjugate of amino-FMISO increased with time, in contrast to those of FMISO, amino-FMISO and the low-molecular-weight fraction (Fig. S2). Conjugation with glutathione makes compounds hydrophilic, which would be expected to decrease cell membrane permeability and retain the conjugate within the hypoxic tumour cells. Thus, the glutathione conjugate of amino-FMISO accumulated progressively in the hypoxic areas of tumours as proposed in Fig. 6. Amino-FMISO accounted for only a small percentage of FMISO metabolites in LC-MS/MS analysis, suggesting that the rate of conversion of hydroxylamino-FMISO to amino-FMISO was slow compared with those of the reactions leading to covalent binding of FMISO to macromolecules and of the production of the glutathione conjugate of amino-FMISO^14^.

It has been assumed that the mechanism of covalent binding of 2-nitroimidazoles to macromolecules involves the binding of the imidazole ring to thiol groups on the macromolecules after reduction of the nitro group in the hypoxic environment (Fig. 6)^15^. Glutathione conjugation is known to also occur through reaction of a thiol group of glutathione with electrophilic compounds^15^. Therefore, the mechanism of FMISO binding to glutathione would be similar to that of 2-nitroimidazoles covalently binding to macromolecules. In addition, in drug discovery research, formation of a glutathione conjugate is regarded as being associated with the ability of agents to covalently bind to macromolecules^16^. Thus, it is reasonable to conclude that glutathione conjugates of amino-FMISO, as well as FMISO covalently bound to macromolecules, accumulate in hypoxic areas of tumours. This may be a general phenomenon also occurring with other 2-nitroimidazole-based compounds.

There are many views regarding the mechanism of FMISO accumulation in hypoxic tumour tissues^3^,^17^,^18^,^19^. Most of these studies did not identify the FMISO-derived compounds that were most contributing to the visualisation of hypoxic areas. This is largely because the distribution of each chemical form cannot be determined with conventional radiochemical analysis. Therefore, we have used IMS to address this issue. IMS can evaluate distribution of each molecule based on its molecular mass. However, conventional IMS has been most frequently applied to the imaging of known molecules because it requires prior structural information to correctly detect distribution of specific analytes in complex tissue samples^20^. In contrast, in this study, we used FT-ICR MS with ultrahigh mass resolution, which can specifically determine the m/z down to the milli mass unit. With this technique, we obtained molecular formulas from mass spectra even without the availability of molecular standards^21^,^22^. This enabled us to identify the glutathione conjugate of amino-FMISO as a metabolite corresponding to the primary contributor to radioactivity observed in ^18^F-FMISO-ARG images.

In histochemical staining of hypoxic regions, pimonidazole is frequently used as a marker of hypoxia. Pimonidazole is also a 2-nitroimidazole based agent, as is FMISO, making it possible that a glutathione conjugate of the reduced form of pimonidazole might also occur in hypoxic tumour tissues. However, during the process of immunohistochemical staining, tissue sections are washed in hydrophilic solvents and the anti-pimonidazole antibody is designed to react mainly with covalently bound forms of pimonidazole^23^. This means that positive staining for pimonidazole could be ascribed to its components covalently bound to macromolecules but not to its low-molecular-weight metabolites, including glutathione conjugates of reduced pimonidazole. In contrast, because FMISO is administered *in vivo*, that is, without “washing” in this manner, its low-molecular-weight metabolites may contribute to acquired PET or ARG images. This makes it necessary to consider whether—in the case of FMISO—the accumulation of low-molecular-weight metabolites really reflects the hypoxic state.

Most compounds targeting hypoxia contain a 2-nitroimidazole structure^5^. Thus, our demonstration, for the first time, of accumulation of the glutathione conjugate of amino-FMISO in hypoxic areas of tumours may help to elucidate accumulation mechanisms of not only FMISO but also other 2-nitroimidazole-based hypoxia imaging probes and other agents. This could contribute to development of more optimised compounds targeting hypoxia.

In conclusion, our IMS study reveals for the first time that a low-molecular-weight metabolite of FMISO, in addition to FMISO derivatives covalently bound to macromolecules, was a significant component of this PET imaging agent’s accumulation in hypoxic tumour tissues.

## Methods

### Chemicals and reagents

All chemicals were commercially available and of the highest available purity. ^18^F-FMISO was obtained from the Hokkaido University Hospital Cyclotron Facility and was synthesised as previously described^24^. Non-radiolabelled FMISO was purchased from FutureChem Co., Ltd. (Busan, Korea). HPLC-grade methanol and acetonitrile were purchased from Kanto Chemical Co., Inc. (Tokyo, Japan). Trifluoroacetic acid (TFA), ammonium hydrogen carbonate and hydrochloric acid were from Wako Pure Chemical Co., Ltd. (Osaka, Japan). 2,5-Dihydroxybenzioc acid (DHB) and indium tin oxide (ITO) glass slides were from Bruker Daltonics (Billerica, MA, USA). Complete protease inhibitor cocktail was from Roche Diagnostics (Basel, Switzerland). Pimonidazole (Hypoxyprobe-1) was purchased from HPI Inc. (Burlington, MA, USA)

### Synthesis of amino-FMISO

Amino-FMISO was synthesised as previously described^13^. Briefly, FMISO (25.2 mg) was dissolved in 2.5 ml methanol and 0.125 ml concentrated HCl was added. After the solution was heated to 90 °C, 500 mg iron (100 mesh) was added and the mixture was refluxed for 30 min. Progress of the reduction process was confirmed by the ninhydrin reaction. The reaction mixture was filtered and then purified by reversed-phase HPLC to obtain amino-FMISO (9.4 mg, 44.5%) using the Shimadzu-HPLC gradient system (LC-20AD system, Shimadzu Corporation, Kyoto, Japan) equipped with an Atlantis T3 column (250 mm × 10 mm, 5 μm, Waters Co., Milford, MA, USA). Chromatographic separation was achieved by gradient elution with a mobile phase composed of 5 mM ammonium hydrogen carbonate (A) and acetonitrile (B). The analytes were eluted by a 1–95% B linear gradient. The total HPLC run time was 20 min at a flow rate of 4 ml/min.

^1^H NMR (DMSO-D6) δ6.85 (s, 1H), 6.79 (s, 2H), 6.71 (s, 1H), 5.57 (d, 1H, *J *= 4.6), 4.42–4.26 (m, 2H,), 3.89–3.85 (m, 2H), 3.78–3.72 (m, 2H): HRMS (m/z) (ESI, pos): [M+H]^+^ calcd. for C_6_H_10_FN_3_O 160.08807; found, 160.08765.

### Tumour xenograft model

Nine-week-old male BALB/c athymic nude mice (Japan SLC, Inc., Hamamatsu, Japan) were housed under a 12-h light/12-h dark cycle with food and water supplied ad libitum. All experimental protocols were approved by the Laboratory Animal Care and Use Committee of Hokkaido University and performed in accordance with the Guidelines for Animal Experiments at the Graduate School of Medicine, Hokkaido University. A human head and neck cancer xenograft model was established using the human head and neck cancer cell line FaDu (American Type Culture Collection, Manassas, VA, USA). This cell line is an established human hypopharyngeal squamous cell carcinoma that grows as an undifferentiated carcinoma in nude mice^25^. The FaDu cells were maintained in Eagle’s Minimum Essential Medium (Sigma-Aldrich, St Louis, MO, USA) supplemented with 10% foetal bovine serum and penicillin (100 u/ml)–streptomycin (100 μg/ml) at 37 °C in a humidified atmosphere of 95% air and 5% CO_2_. The FaDu cells (5 × 10^6^ cells) suspended in 100 μl phosphate-buffered saline were inoculated subcutaneously into the right flank of each mouse. Further experiments were performed after a 2-week tumour growth period. All animal manipulations were performed using sterile techniques.

### Animal experiments

For experiments measuring covalent binding of FMISO to macromolecules and radio-HPLC analysis, ^18^F-FMISO (270–430 MBq) was injected into the tumour-bearing mice via the tail vein and, 4 h later, mice were sacrificed and tumour tissues immediately excised.

For IMS and ARG experiments, a mixture of ^18^F-FMISO (10 MBq) and non-labelled FMISO (550 mg/kg), dissolved in aqueous solution (dimethylacetamide/saline = 1:3), was injected into the tumour-bearing mice via the tail vein. Two hours after FMISO injection, pimonidazole (100 mg/kg) was intravenously injected into the same mice. These mice were sacrificed at 4 h after FMISO administration and tumour tissues were immediately excised and frozen in dry ice powder. (An IMS study performed with samples collected at 2 h, instead, showed a similar distribution, but a weaker signal (Supplementary Figure S3) confirming our choice of the 4 h time point. In this experiment, pimonidazole was intravenously injected into the mice 1 hour after FMISO injection.) Serial cross sections at a 10-μm thickness were immediately cut and thaw-mounted on a glass slide using a CM3050-Cryostat (Leica Microsystems; Wetzlar, Germany). For the biodistribution study, blood, heart, liver, kidney, muscle, and bone were harvested along with tumour samples, weighed and counted for radioactivity in a gamma counter (Wizard 3′′ 1480 Automatic Gamma Counter; PerkinElmer, Inc., Waltham, MA, USA). These samples were harvested at 2 and 4 h after FMISO administration.

### Determining covalent binding of FMISO to macromolecules

The tumour of each mouse was weighed, suspended in phosphate-buffered saline with protease inhibitor cocktail (4 ml/g of tissue) and crushed at 3,000 rpm for 1 min with 1- and 3-mm-diameter zirconia beads using a Micro Smash™ instrument (Tomy Seiko Co., Ltd., Tokyo, Japan) at 4 °C. The homogenised samples were twice extracted with methanol and radioactivity in supernatants and precipitates was determined by counting aliquots in a gamma counter. These samples were considered the low-molecular-weight and macromolecule-bound fractions, respectively.

### Radio-HPLC analysis of low-molecular-weight fractions of tumour homogenates

For metabolite analysis, 10-μl aliquots of concentrated extracts from tumour homogenates were chromatographed using a Shimadzu HPLC gradient system monitored at 220 nm (LC-20AD system, Shimadzu Corporation, Kyoto, Japan) equipped with an XBridge C18 column (150 mm × 4.6 mm, 5 μm, Waters). Chromatographic separation was achieved by gradient elution with a mobile phase composed of 5 mM ammonium hydrogen carbonate (A) and acetonitrile (B). The analytes were eluted with a 1–95% B linear gradient. The total HPLC run time was 20 min at a flow rate of 1 ml/min. Consecutive 0.5-min HPLC fractions were collected from the elution and radioactivity of fractions was measured in a gamma counter. Solutions of FMISO and amino-FMISO were employed as standards.

### Autoradiography

Tumour cryosections were exposed to a phosphor image plate (Fuji Imaging Plate BAS-SR 2025, Fuji Photo Film Co., Ltd., Tokyo, Japan) together with a set of calibrated standards^26^ for 12 h. After each exposure, the imaging plate was scanned with a computerised imaging analysis system (FLA 7000 Bio-Imaging Analyzer; Fuji Photo Film Co.) and images were analysed by Multi Gauge V3.2 (Fuji Photo Film Co.). To evaluate radioactivity derived from the fraction of FMISO covalently bound to macromolecules, the serial sections were washed with ethanol and tris-buffered saline once and three times, respectively, and then immersed in ethanol for 3 min to remove extractable ^18^F-FMISO. After sections were dried, ARG images were acquired as described above.

### Immunohistochemical staining of pimonidazole

Tumour sections mounted on immuno-coat micro slides (Muto Pure Chemicals Co., Ltd., Tokyo, Japan) were stained for pimonidazole to assess tumour hypoxia. After rehydration, endogenous peroxidase activity was blocked for 10 min with 0.3% hydrogen peroxide. Slides were next incubated with Hypoxyprobe-1 MAb1 (HPI Inc., Burlington, BA, USA) for 30 min at 37 °C, followed by incubation with biotin-conjugated F(ab’)_2_ for 15 min at 37 °C. The bound antibody complex was then visualised by incubation with streptavidin and 3,3′-diaminobenzidine tetrahydrochloride. Images of the tumour sections stained by the anti-pimonidazole antibody were captured under a microscope (Biozero BZ-8000; Keyence Co., Osaka, Japan).

### Sample preparation for MALDI-IMS

Tumour sections were placed onto indium tin oxide-coated glass slides and stored at −20 °C until analysis. Prior to matrix coating and mass spectrometric analysis, slides were placed in a vacuum desiccator for 15 min at room temperature and optical images were acquired using a scanner (GT-X820; Seiko Epson Corporation, Nagano, Japan) to identify the location of each tissue. Sections were then coated with the matrix solution (30 mg/ml DHB dissolved in 1:1, v/v methanol–water containing 0.2% TFA or 20 mg/ml DHB dissolved in 3:17, v/v methanol–water containing 0.2% TFA) using an ImagePrep™ automated device using vibrational vaporisation technology (Bruker Daltonics Inc., Billerica, MA, USA).

### MALDI-IMS study

IMS analysis was performed using a 7T Bruker solariX XR MALDI FT-ICR MS (Bruker Daltonics Inc.) equipped with a SmartBeam II UV laser. Data were acquired and analysed using fleximaging for imaging experiments (Bruker Daltonics Inc.). The laser energy and the raster step size were set at 40% and 150 μm, respectively. Analytes were detected in the positive-ion mode. FMISO was detected by the product ion scans of the [M +H]+ ion of FMISO (m/z 190). The collision energy was 10%. The m/z 164.0673 fragment ion was generated on the tissue. This ion was also observed as the derivative of authentic FMISO.

### LC-MS/MS analysis

To obtain product ion spectra and determine concentrations of FMISO and its metabolites, tumour homogenates were injected into the LC-MS/MS system. An Acquity ultra-performance LC (UPLC) system (Waters Co., Milford, MA, USA) with a triple quadrupole mass spectrometer (API 5000™, AB Sciex, Foster City, CA, USA) was used for LC-MS/MS analysis. The LC-MS/MS system was controlled by Analyst 1.4.2 (AB Sciex) software. Chromatographic separation was performed using a YMC-Triart C18 column (50 × 2 mm, 1.7 μm, YMC Co., Ltd., Kyoto, Japan) at 25 °C. Separation was achieved by gradient elution with mobile phase composed of 15 mM ammonium hydrogen carbonate (A) and acetonitrile (B). The analytes were eluted by a 1–95% B linear gradient. The total UPLC run times were 20 and 3 min, at flow rates of 0.2 and 0.5 ml/min for obtaining product ion spectra and determining concentrations, respectively. The autosampler compartment temperature was 4 °C. ESI was performed in the positive-ion mode.

## Additional Information

**How to cite this article**: Masaki, Y. *et al.* The accumulation mechanism of the hypoxia imaging probe “FMISO” by imaging mass spectrometry: possible involvement of low-molecular metabolites. *Sci. Rep.* **5**, 16802; doi: 10.1038/srep16802 (2015).

## Supplementary Material

Supplementary Information
