# Supplementary material for: The accumulation mechanism of the hypoxia imaging probe “FMISO” by imaging mass spectrometry: possible involvement of low-molecular metabolites
Source: Sci Rep. 2015 Nov 19;5:16802. doi: 10.1038/srep16802 (PMC4652161; doi:10.1038/srep16802)
Supplement: Supplementary Information [file srep16802-s1.pdf]

The accumulation mechanism of the hypoxia imaging probe “FMISO” by imaging mass spectrometry: possible involvement of low-molecular metabolites

Yukiko Masaki[1)], Yoichi Shimizu[2-4)] *, Takeshi Yoshioka[1)], Yukari Tanaka[5)], Ken-ichi Nishijima[2,3)], Songji Zhao[3)], Kenichi Higashino[1)], Shingo Sakamoto[5)], Yoshito Numata[1)], Yoshitaka Yamaguchi[5)], Nagara Tamaki[3)], Yuji Kuge[2,3)]

[1)]Shionogi Innovation Center for Drug Discovery, Discovery Research Laboratory for Innovative Frontier Medicines, Shionogi & Co., Ltd., Sapporo 001-0021, Japan,

[2)]Central Institute of Isotope Science, Hokkaido University, Sapporo 060-0815, Japan,

[3)]Graduate School of Medicine, Hokkaido University, Sapporo 060-8638, Japan

[4)]Faculty of Pharmaceutical Sciences, Hokkaido University, Sapporo 060-0812, Japan,

[5)]Shionogi Pharmacological Research Center, Research Laboratory for Development, Shionogi & Co., Ltd., Osaka 561-0825, Japan.

Supplementary information

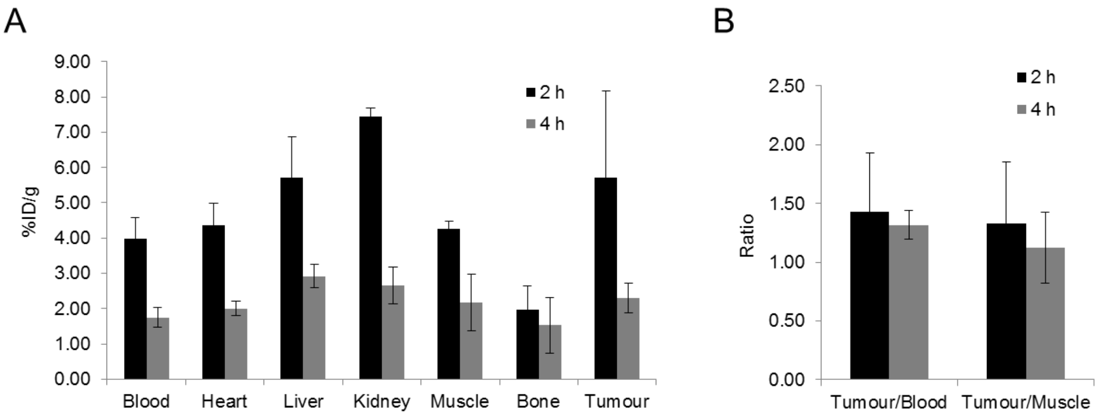

Supplementary Figure 1. Biodistribution of ^{18}F-FMISO in tumour-bearing mice 2 and 4 h after administration of ^{18}F-FMISO. Data are means ± s.d. (n=3–4).

A: The radioactivity in tissues of mice after injection of ^{18}F-FMISO. Tissue radioactivity is expressed as % injected dose (ID) per gram of tissue.

B: Tumour-to-blood or tumour-to-muscle radioactivity ratio of ^{18}F-FMISO.

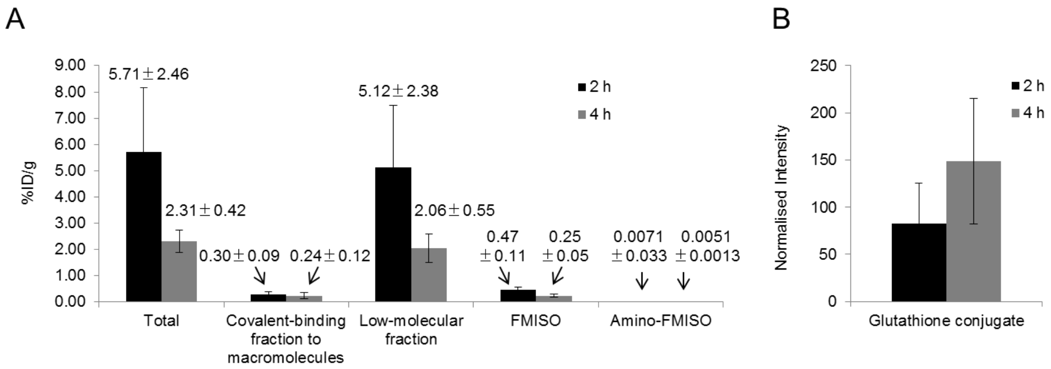

Supplementary Figure 2. Distribution of total radioactivity, FMISO and its metabolites 2 and 4 h after administration of ^{18}F-FMISO. Data are expressed as means ± s.d. (n=3–4).

A: Uptake of total radioactivity, FMISO and its metabolites in tumour. Each radioactivity value is expressed as % ID per gram of tumour tissue.

B: Relative uptake of glutathione conjugate of amino-FMISO in tumour. The value is expressed as normalised intensity in which the signal intensity is normalised to the injected dose.

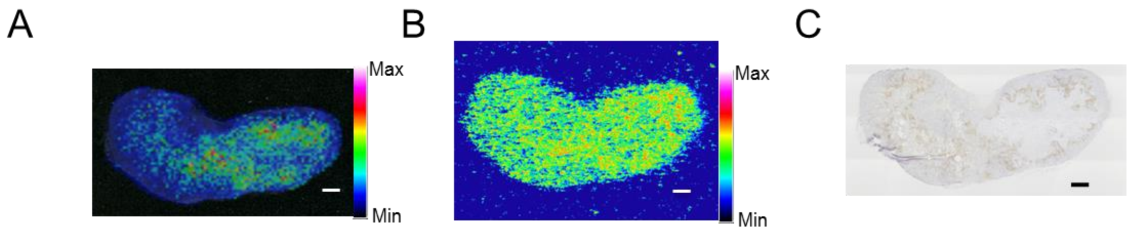

Supplementary Figure 3. Representative mass spectrometric image of glutathione conjugate of amino-FMISO, ARG and pimonidazole staining in mouse tumour 2 h after administration of ^{18}F-FMISO. Scale bar represents 1 mm.

A: Mass spectrometric images of m/z 465.157 representing glutathione conjugate of amino-FMISO.

B: ARG image of the serial tumour section.

C: Immunohistochemical staining for pimonidazole.
